# Ultra-Trace Elemental and Isotopic Quantification for Neonatal Nutrition Studies

**DOI:** 10.6028/jres.093.064

**Published:** 1988-06-01

**Authors:** L. J. Moore, J. E. Parks, M. T. Spaar, D. W. Beekman, E. H. Taylor, V. Lorch

**Affiliations:** Atom Sciences, Inc., 114 Ridgeway Center, Oak Ridge, TN 37830; Intensive Care Nursery, Department of Pediatrics, University of Tennessee, Knoxville, TN 37920

Trace element accumulation in the human fetus occurs primarily in the third trimester of pregnancy, and premature birth interrupts this process. A study of zinc in low birth weight infants indicates that the fetus accrues 310 *μ*g of Zn daily at the 30th week, increasing to 590 *μ*g daily by the 36th week of gestation [1]. Similarly, the human fetus accumulates 80–90 *μ*g of Cu/kg/day between 28 and 36 weeks, and by 40 weeks the fetus has accumulated almost 20 mg of Cu, one half of which is in the liver [2]. These and other essential trace elements play vital roles in the adult. Zinc is required for the synthesis of DNA, RNA, and protein, and as the zinc metallo-enzyme, regulates growth through DNA polymerase, RNA polymerase and thymidine kinase. References to these effects have been summarized [3]. Significant progress has been achieved recently using stable isotope tracers to assess the metabolic and nutritional roles of Ca [4], Zn [5], Se [6], and Mg [7] in adults and healthy infants. Techniques used for these studies include mass spectrometry, using electron impact ionization of metal chelates or thermal ionization of inorganic species. Trace elements at higher concentrations or in large samples have been determined with techniques such as atomic absorption (AA) or emission spectroscopy that do not address measurement needs for trace element and isotope tracer determinations in premature infants, healthy newborns, children, pregnant women, and other adults.

Little is known about the presence and function of trace and ultratrace elements in pre-term infants whose birth weight is 500–2000 g. Only microliters of blood are available, and trace elements can occur in the blood at levels of parts per billion (ng/g) or lower. For a trace element level of 1 ppb, the total amount of the element available is 100 pg, or assuming vanadium, only 10^12^ atoms. Clearly a new and comprehensive method is required to access elemental and isotopic information at these levels. Resonance ionization spectroscopy (RIS) is being used to solve these problems.

RIS utilizes a source of tunable laser radiation that is resonant with a specific atomic energy level. In the simplest two photon RIS scheme (fig. 1, scheme 1), a second photon of the same energy promotes the excited electron to the ionization continuum, forming an atomic ion. This simple two photon process can be used to ionize approximately fifty elements, but a series of RIS schemes has been proposed by Hurst et al., to ionize all elements except He and Ne (fig. 1) [8].

Resonance ionization advantages are threefold: 1) sensitivity, 2) selectivity, and 3) generality. Ionization *sensitivity* is achieved by saturating the photon absorption cross section of the energy level such that every atom crossing the active ionizing laser volume is ionized with unit probability. Elemental ionization *selectivity* is achieved through wavelength tunability. By selecting appropriate non-overlapping wavelengths, elements can be measured quantitatively in the presence of 10^6^–10^12^ of other elements. Resonance ionization is a *general* ionization process as described above.

## Experimental

The experimental design was structured toward the longer term research goal: to provide for the neonatologist a total multi-elemental and isotopic diagnostic analysis using only a few microliters of blood. Thus, the chemical separations must be minimal, simple, and capable of providing a group separation in a form amenable to RIS analysis. We selected electrodeposition as an initial separation process and were successful in depositing nanogram or picogram quantities of Cu, Mo, Se, and V singly or simultaneously onto a high purity gold substrate directly from aqueous solution. Cu and Mo were deposited individually onto gold from processed serum samples and their concentrations determined by isotope dilution. To separate copper, the serum samples were wet-ashed with a nitric-perchloric acid mixture, evaporated to dryness, adjusted to the appropriate acidity, and the copper electrodeposited. To separate molybdenum, the sample was wet-ashed as above but the molybdenum was deposited from alkaline solution.

**Figure 2:**
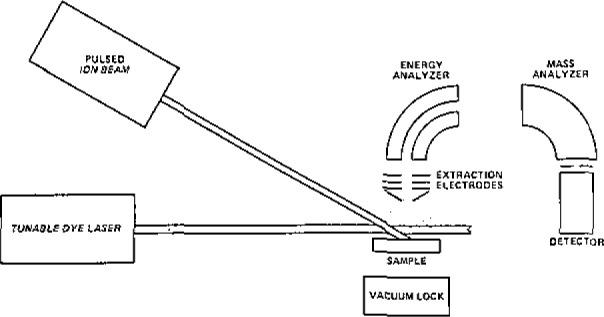
The sputter-initiated resonance ionization spectrometer (SIRIS) system used for the analytical determinations reported here. Condensed phase samples are atomized with an energetic argon primary ion beam ionized with a multi-color tunable dye laser system, and the ions are energy and mass analyzed with a double focusing mass spectrometer.

Following the multicomponent analysis theme, the laser and optical systems in Atom Sciences’ Sputter-Initiated Resonance Ionization Spectrometer (SIRIS) (fig. 2) were modified to permit access to Cu, Zn, Mo, Se, and V within a single tunable laser dye range. Samples on gold foil were atomized by ion sputtering and the diluted isotope ratios were determined using a double-focusing mass spectrometer. The mass spectrometer was calibrated using gravimetrically prepared mixtures of isotopically natural copper and enriched ^65^Cu.

## Results and Discussion

**Figure 3:**
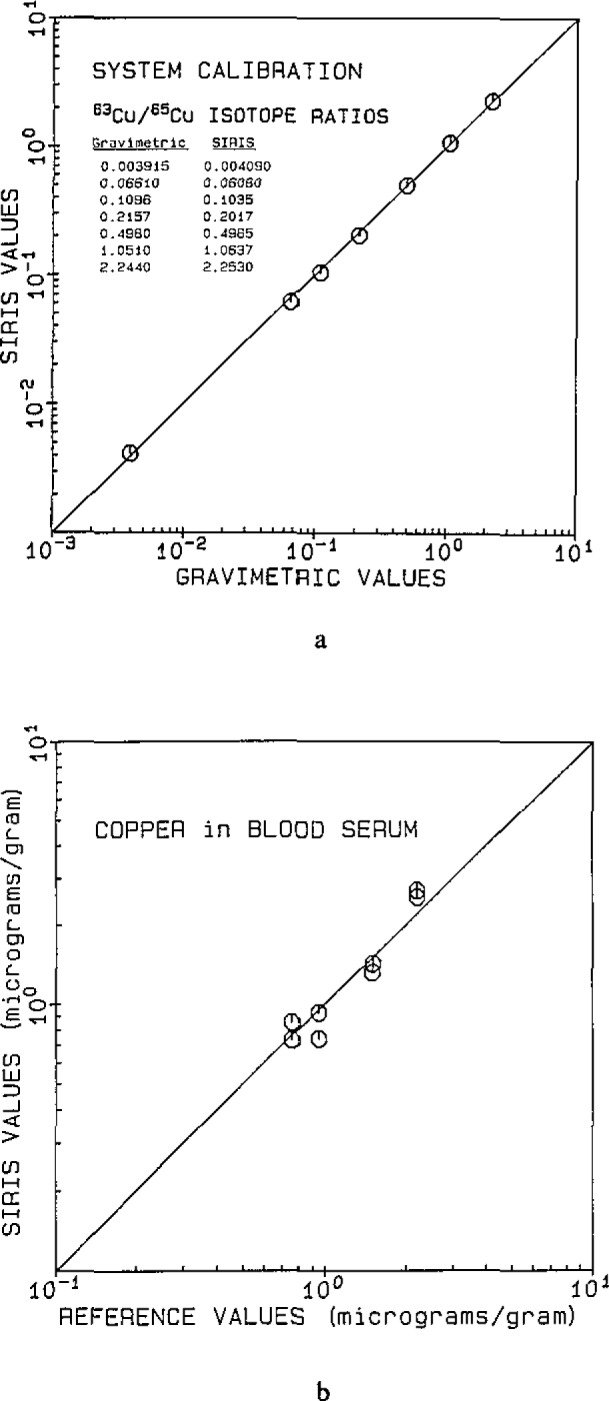
a. A calibration of the SIRIS system using ^63^Cu/^65^Cu isotope ratio standards, prepared by mixing natural copper (^63^Cu/^65^Cu=2.244) and the ^65^Cu enriched isotope (^63^Cu/^65^Cu=0.0038). The relative standard deviation observed for the calibration was 4.7%; b. a plot of copper concentrations in bovine (RM 8419) and human serum determined with SIRIS, versus concentrations determined by flame AA using 2–3 mL samples. Serum sample sizes used for SIRIS were nominally 100 *μ*L and were separate duplicate determinations. The lowest concentration reported here was for bovine serum (RM 8419), and was 0.76 *μ*g/g compared to the suggested value of 0.73 *μ*g/g (Ref. [9]).

The results of the SIRIS calibration are illustrated in figure 3a. The data points cover an isotope ratio range of nearly 600, from natural copper (^63^Cu/^65^Cu=2.244) to the separated ^65^Cu isotope (^63^Cu/^65^Cu=0.0038). This range encompassed the anticipated ^63^Cu/^65^Cu range for the isotope dilution samples, nominally 0.05 to 0.4. The deviation of each observed ratio from its gravimetric value was summed over all the ratios, yielding a relative standard deviation (RSD) of 4.7%. Isotope ratio precisions internal to an analysis were typically 1–2%, with attendant standard errors of the mean of 0.2%.

The concentration of copper was determined in separately processed duplicate samples of human serum and plotted in figure 3b versus analyses of the same (but larger) samples by flame AA. The RIS sample sizes were 80–200 *μ*L, and an overall accuracy of ± 10% was estimated, which included contributions from inhomogeneities, RIS methodology and the flame AA results using larger (2–3 mL) aliquots. Using isotope dilution with ^100^Mo, Mo was determined in high purity reagents and serum. Concentrations of Mo in reagents, pg/g, were: H_2_O, 13; HNO_3_, 44; HCIO_4_, 30; and an Mo concentration of 19 ng/g was determined for a 250 *μ*L aliquot of Bovine Serum (RM 8419), compared to the suggested value of 16 ng/g [9].

Using SIRIS, sensitivities have been observed for Mo isotopes at the few pg level. Overall ionization and instrumental transmission efficiencies indicate potential absolute sensitivites of a few thousand atoms (⩽10^−9^ monolayer). The RIS elemental and isotopic analysis technology is expected to be generally applicable in medicine and nutrition for metabolic and diagnostic studies.

## Figures and Tables

**Figure 1:**
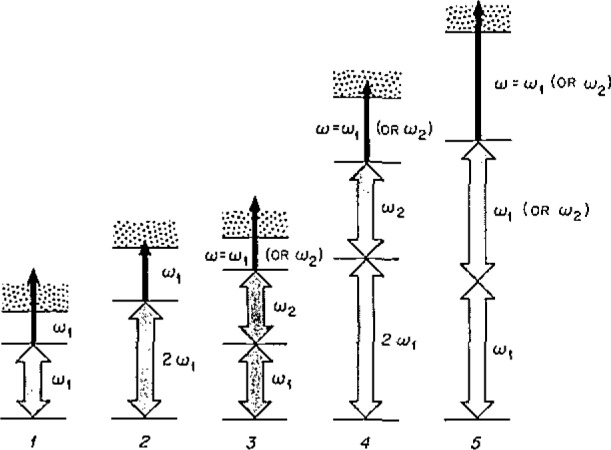
A schematic illustration of five resonance ionization processes that are possible using commercially available lasers (taken from Ref. [8]). RIS of Mo is by a generalization of scheme 1, where *ω*_1_ refers to a frequency doubled laser beam. Analogously, RIS of Cu is by a generalization of scheme 3, where *ω*_1_=*ω*_2_=frequency doubled beams, although ionization is by *ω*_3_=IR.
